# Late radiation-related lymphopenia after prostate stereotactic body radiation therapy plus or minus supplemental pelvic irradiation

**DOI:** 10.3389/fonc.2024.1459732

**Published:** 2024-11-21

**Authors:** Kelly Gaudian, Min Jung Koh, Min Ji Koh, Peter Jermain, Irfan Khan, Diya Kallam, Zach Lee, Ryan R. Collins, Zoya Zwart, Malika Danner, Alan Zwart, Deepak Kumar, Michael B. Atkins, Simeng Suy, Sean P. Collins

**Affiliations:** ^1^ Washington University School of Medicine in St. Louis, St. Louis, MO, United States; ^2^ Department of Radiation Medicine, Georgetown University Hospital, Washington, DC, United States; ^3^ College of Medicine, George Washington University, Washington, DC, United States; ^4^ Department of Radiation Oncology, University of South Florida (USF) Health Morsani College of Medicine, Tampa, FL, United States; ^5^ Biotechnology Research Institute, North Carolina Central University, Durham, NC, United States; ^6^ Department of Oncology, Lombardi Comprehensive Cancer Center, Georgetown University Medical Center, Washington, DC, United States

**Keywords:** lymphopenia, SBRT, supplemental pelvic radiation therapy, prostate cancer, radiation related toxicities

## Abstract

**Introduction:**

Prior studies suggest lymphopenia following radiation therapy may impact toxicity and cancer control. Chronic radiation-related lymphopenia (RRL) has been noted in prostate cancer patients treated with conventionally fractionated pelvic radiation therapy. The impact of utilizing hypofractionated high integral dose therapies such as stereotactic body radiation therapy (SBRT) on RRL is less well characterized. This prospective study sought to evaluate the impact of prostate SBRT plus or minus supplemental pelvic nodal radiation (PNI) on RRL.

**Methods:**

Between 2012 and 2023, serial serum absolute lymphocyte counts (ALCs) were measured in 226 men treated at MedStar Georgetown with robotic SBRT using the CyberKnife® (CK) (36.25 Gy in 5 fractions) alone or CK (19.5 Gy in 3 fractions) followed by supplemental PNI using VMAT (37.5–45.0 Gy in 15–25 fractions) per an institutional protocol (IRB#: 2012-1175). Baseline ALC (k/μL) was measured 1–2 hours prior to robotic SBRT and at each follow-up appointment (1, 3, 6, 9, 12, 18, and 24 months post-treatment). Lymphopenia was graded using the CTCAEv.4: Grade 1 (0.8-1.0 k/μL), Grade 2 (0.5-0.8 k/μL), Grade 3 (0.2-0.5 k/μL) and Grade 4 (<0.2 k/μL). To compare two different treatment groups, the Wilcoxon signed-rank test was used. A p-value of < 0.05 determined statistical significance.

**Results:**

Of 226 patients (SBRT alone: *n* = 169, SBRT + PNI: *n* = 57), the median age was 72 years and 45% of patients were non-white. Baseline lymphopenia was uncommon and of low grade. In the SBRT alone group, the baseline ALC of 1.7 k/μl decreased by 21% to 1.4 k/μL at 3 months and then stabilized. 38% of these men experienced lymphopenia in the two years following SBRT, however, no patient presented with Grade 3 lymphopenia. Patients who received SBRT + PNI had a lower baseline ALC (1.5 k/μl), and a significantly greater decrease in ALC relative to individual baseline value throughout the 2-year follow-up period, decreasing by 57% to 0.6 k/μL at 3 months and recovering to a 36% decrease from baseline (1.0 k/μL) at 24 months. Notably, 12% of the men treated with SBRT + PNI experienced Grade 3 lymphopenia. No patient in either cohort experienced Grade 4 lymphopenia.

**Discussion:**

The low incidence of high-grade lymphopenia within this elderly patient population further supports the safety of prostate SBRT plus or minus PNI for the treatment of prostate cancer. However, RRL was more severe when PNI was utilized. The effect of SBRT and PNI on lymphocytes in prostate cancer patients could act as a model for other cancers, specifically those involving treatment with immunomodulatory agents. Future studies should focus on the clinical implications of RRL and the effects of specifically irradiating lymphoid tissues on lymphocyte biology.

## Introduction

Prior studies suggest lymphopenia following radiation therapy may impact toxicity and cancer control. Lymphocytes are highly radiosensitive, and exposure even to low doses of radiation therapy (RT) (<2 Gy) poses a significant risk of lymphocyte death (1). Given their role in mediating the immune response to cancer, radiation-related lymphopenia (RRL) has the potential to impact disease-specific survival in patient cohorts treated with radiation regimens (1). In a study of solid tumors of the brain, head and neck, thorax, and abdomen, lymphopenia cases categorized as Grade 3 or higher by the CTCAEv.4 following radiation therapy were common and shown to negatively impact overall survival rates (2–4).

In prostate cancer, chronic RRL has been noted in patients treated with conventionally fractionated pelvic radiation therapy (5). More specifically, pelvic nodal irradiation (PNI) was found to be correlated with RRL in patients with prostate cancer, and increased treatment volume was positively associated with a higher RRL burden (6, 7). The absolute lymphocyte count (ALC) nadir typically occurs during or immediately after radiation. The effect of utilizing hypofractionated high integral dose therapies in prostate cancer, such as stereotactic body radiation therapy (SBRT), on RRL incidence and severity is unknown.

SBRT precisely targets tumors with intrafractional image guidance in up to five fractions (8). When compared to chemotherapy, patients treated for pancreatic cancer with SBRT experienced a lower incidence of lymphopenia overall, suggesting that differences in treatment regimen can have a significant effect on lymphocyte levels (9). When compared to conventionally fractioned radiation therapy (CFRT) for pancreatic cancer, SBRT was again found to result in a lower lymphopenia incidence, with acute Grade 3/4 lymphopenia of 6% for SBRT versus 38% for CFRT (10, 11).

As shown in the literature, the occurrence of more severe lymphopenia can potentially impact the efficacy of current and future courses of treatment. In a patient cohort treated with SBRT for lung cancer, lymphopenia following SBRT was found to be associated with a poorer prognosis for survival (12). This study identified low pre-treatment lymphocyte counts, longer duration of treatment, and higher mean dose as predictors for the possibility of developing lymphopenia post-SBRT (12). Given this, delivering doses of radiation to avoid lymphocyte-rich structures has been thought to improve outcomes in solid malignancies (9).

Radiation therapy has been increasingly utilized in combination with immunotherapy, and lymphopenia has been found to be an indicator of poor prognosis post-treatment with immunotherapy (13). Patients who developed lymphopenia after radiation therapy and later received immunotherapy treatment, had worse outcomes when compared to patients who did not experience RRL (13). Further characterizing the impacts of RRL from varied radiation treatment types could be used to improve outcomes when utilizing RT in combination with other classes of agents. This prospective study sought to evaluate the impact of prostate SBRT plus or minus supplemental pelvic nodal radiation (PNI) on chronic RRL incidence and severity.

## Methods

Between 2012 and 2023, serial serum ALCs were measured in 226 men treated at MedStar Georgetown with robotic SBRT using the CyberKnife^®^ (CK) (36.25 Gy in 5 fractions) alone or CK (19.5 Gy in 3 fractions) followed by supplemental PNI using VMAT (37.5–45.0 Gy in 15–25 fractions) per an institutional protocol (IRB#: 2012-1175) (14, 15). Patients were not randomized to treatment groups.

Four to six gold fiducials were placed into the prostate. Seven days after fiducial placement, patients underwent magnetic resonance (MR) imaging followed shortly thereafter by a thin-cut computed tomography (CT) scan. Fused CT and MR images were used for treatment planning. The clinical target volume (CTV) included the prostate and the proximal seminal vesicles (to the point where the seminal vesicles separate). The planning target volume (PTV) equaled the CTV expanded 3 mm posteriorly and 5 mm in all other dimensions. The prescription dose was 36.25 Gy to the PTV delivered in five fractions of 7.25 Gy, corresponding to a tumor equivalent dose in 2Gy fractions (EQD2) of approximately 85–90 Gy assuming an α/β ratio of 1.5.

Treatment plans were composed of hundreds of pencil beams using variable-sized circular collimators (Iris, Accuray) to generate highly conformal plans using Multiplan^®^ or Volo^®^ (Accuray Inc., Sunnyvale, CA) inverse treatment planning. Plans were inhomogeneous by design to minimize dose to adjacent critical structures. Radiation was delivered every other day to a mean prescription isodose line of 83% in 5 approximately 35-min treatments. On average, 100–200 beams were employed. Target position was verified every 30–60 s during treatment using paired, orthogonal kV images (16).

For the SBRT + PNI patients, the CTV1 included the prostate, areas of radiographic extracapsular extension, and seminal vesicles proximal to the point of separation. The SBRT PTV1 was equal to the CTV1 expanded 3 mm posteriorly and 5 mm in all other dimensions. The prescription dose was 19.5 Gy to PTV1 delivered in 3 fractions of 6.5 Gy over 3–5 days. Following SBRT, intensity-modulated radiation treatment (IMRT) treatment was initiated the following week. PTV2 included the prostate, entire seminal vesicles, and RTOG consensus pelvic nodes with a margin of 1.0 cm around CTV1, except at the rectal interface where a margin of 0.5 cm was added. The superior extent of disease was the sacrum-L5 junction. Daily doses of 1.8 to 2.5 Gy were delivered to PTV2 5 days a week to a total dose of 37.5–45 Gy in 15–25 fractions. One hundred percent of PTV2 received at least 95% of the prescription dose, and 5% of the volume received no more than 105% of the prescription dose.

Baseline ALC (k/μl) was measured on a complete blood count (CBC) panel 1–2 hr prior to the first fraction of robotic SBRT and at each follow-up appointment (1, 3, 6, 9, 12, 18, and 24 months). Lymphopenia was graded using the CTCAEv.4: Grade 1 (0.8–1.0 k/μl), Grade 2 (0.5–0.8 k/μl), Grade 3 (0.2–0.5 k/μl), and Grade 4 (<0.2 k/μl). To compare two different treatment groups, the Wilcoxon signed-rank test was used. A *p*-value of <0.05 determined statistical significance.

## Results

Demographic and clinical patient characteristics are presented in **Table 1**. Of 226 patients (SBRT alone: *n* = 169, SBRT + PNI: *n* = 57), the median age was 72 years, and 45% of patients were non-White. High-risk prostate cancer and utilization of androgen deprivation therapy (ADT) were more common in those who received PNI (*p* < 0.01) (**Table 1**). The median pelvic nodal PTV volume was 697.4 ± 129.8 cc.

**Table 1:** Demographic and clinical characteristics.

Characteristics	No. (%)			P value
	All (n = 226)	SBRT alone (n = 169)	SBRT + PNI (n = 57)	
Age (y), Mean ± SD	72±7.9	71±7.6	73±8.5	0.1^a^, 0.1^b^
<60	19 (8)	14 (8)	5 (9)	
60-69	70 (31)	55 (33)	15 (26)	
70-79	111 (49)	86 (51)	25 (44)	
>80	26 (12)	14 (8)	12 (21)	
Race				0.6^b^
White	127 (56)	97 (57)	30 (53)	
Black	75 (33)	56 (33)	19 (33)	
Other	24 (11)	16 (9)	8 (14)	
Risk Group				<0.01^b^
Low	16 (7)	16 (9)	0 (0)	
Intermediate	128 (57)	124 (73)	4 (7)	
High	81 (36)	28 (17)	53 (93)	
Recurrent	1 (<1)	1 (1)	0 (0)	
ADT				<0.01 ^b^
Yes	120/211 (57)	91/157 (58)	0 (0)	
No	91/211 (43)	66/157 (42)	54/57 (100)	

a P-values for the comparison between patients who were on SBRT alone or on SBRT and PNI using t-test and chi-square test for normally distributed continuous, and categorical variables, respectively.

b P-values based on Fisher's exact test due to some small cell counts.

**Figure 1:**
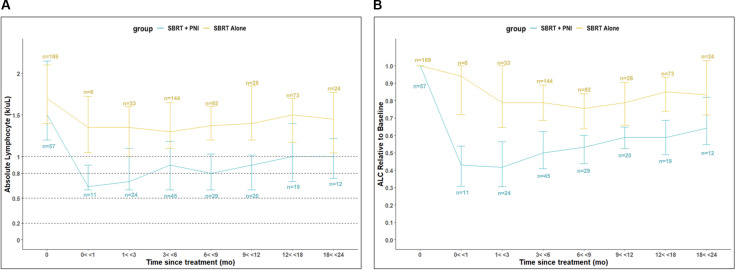
Absolute Lymphocyte Count (ALC) Relative to Baseline in SBRT vs. SBRT + PNI Patients. **(A)** Abosolute Lymphocyte Count (ALC) (k/uL). **(B)** ALC Relative to Baseline. An absolute number (n) of patients is shown above or below respective timepoints, also shown in **Supplementary Table 1**.

Baseline lymphopenia was uncommon and of low grade (**Table 2**). For the SBRT alone group, the baseline ALC of 1.7 k/μl decreased by 21% to 1.4 k/μl at 3 months and then stabilized (**Table 3A**; **Figure 1**). In the two years following SBRT, 23% (*n* = 38) of these men experienced lymphopenia (Grade 1: *n* = 25; Grade 2: *n* = 13) (**Table 2**). No SBRT alone patient presented with a Grade 3 or Grade 4 lymphopenia over the 24-month time course studied (**Table 2**).

**Table 2:** Lymphopenia (CTCAEv.4) categories: toxicities by grade.

Lymphopenia Grade	No. (%)			
	Baseline (t = 0)	0<t<24 (months)	SBRT Alone	SBRT + PNI
**No Toxicity** (>1.0)	210 (93)	141 (62)	131 (78)	10 (18)
**Grade 1** (0.8-1.0)	11 (5)	38 (17)	25 (15)	13 (23)
**Grade 2** (0.5 - 0.8)	4 (2)	40 (18)	13 (8)	27 (47)
**Grade 3** (0.2-0.5)	1 (<1)	7 (3)	0 (0)	7 (12)

**Table 3:** Median (IQR) plots.

A. Absolute Lymphocyte Count (k/μL)			
Time point (months)	Median (IQR) (k/µL)		P value (Wilcoxon rank sum test)
	SBRT Alone	SBRT + PNI	
**0**	1.7 (1.4-2.1)	1.5 (1.2-2.2)	0.2
**0≤ t <1**	1.4 (1.1-1.7)	0.6 (0.6-0.9)	0.007
**1≤ t <3**	1.4 (1.0-1.6)	0.7 (0.6-1.1)	<0.0001
**3≤ t <6**	1.3 (1.1-1.7)	0.9 (0.6-1.2)	<0.0001
**6≤ t <9**	1.4 (1.2-1.6)	0.8 (0.6-1.0)	<0.0001
**9≤ t <12**	1.4 (1.2-1.9)	0.9 (0.6-1.0)	<0.0001
**12≤ t <18**	1.5 (1.2-1.7)	1.0 (0.7-1.4)	<0.001
**18≤ t <24**	1.5 (1.0-1.8)	1.0 (0.7-1.2)	<0.001
B. ALC Relative to Baseline			
Time point (months)	Median (IQR)		P value (Wilcoxon rank sum test)
	SBRT Alone	SBRT + PNI	
**0**	1	1	–
**0≤ t <1**	0.94 (0.72-1.0)	0.43 (0.31-0.54)	0.002
**1≤ t <3**	0.79 (0.65-1.0)	0.42 (0.31-0.56)	<0.0001
**3≤ t <6**	0.79 (0.69-0.89)	0.50 (0.41-0.62)	<0.0001
**6≤ t <9**	0.76 (0.64-0.84)	0.53 (0.44-0.60)	<0.0001
**9≤ t <12**	0.79 (0.66-0.90)	0.59 (0.52-0.65)	<0.001
**12≤ t <18**	0.85 (0.74-0.93)	0.59 (0.49-0.69)	<0.0001
**18≤ t <24**	0.83 (0.72-1.03)	0.64 (0.55-0.82)	0.01

SBRT + PNI had significantly lower ALC and a greater decrease in ALC relative to individual baseline values throughout the 2-year follow-up period. The baseline ALC of 1.5 k/µl decreased by 57% to 0.6 k/µl at 3 months and recovered to a 36% decrease from baseline (1.0 k/µl) at 24 months (**Tables 3A, B**; **Figure 1**). In the 2 years following SBRT, 82% (*n* = 47) of these men experienced lymphopenia (Grade 1: *n* = 13; Grade 2: *n* = 27; Grade 3: *n* = 7) (**Table 2**). Notably, 12% of the men treated with SBRT + PNI experienced Grade 3 lymphopenia. No patient experienced Grade 4 RRL over the 24-month follow-up period (**Table 2**).

## Discussion

This study demonstrates that within our elderly patient cohort, the low incidence of high-grade lymphopenia further supports the safety of prostate SBRT, plus or minus PNI, for the treatment of prostate cancer. However, RRL was more severe when PNI was utilized. When compared to SBRT alone, patients who received SBRT + PNI experienced a significantly greater decrease in ALC relative to baseline throughout the follow-up period. This result suggests that in combination with SBRT, supplemental PNI increases the severity of lymphopenia post-RT.

Patients treated with SBRT alone experienced a maximum decline to the lowest median ALC by 6 months post-treatment, after which recovering to a median of 1.5 k/µl (88% of time point 0 value) at 12 months and plateauing through 24 months. This reflects a nearly full recovery to baseline lymphocyte populations after 12 months. Additionally, median ALC values in this group are graded as “No Toxicity” across the entire 24-month recovery period. Patients treated with SBRT + PNI experienced a maximum decline in ALC by 1 month post-treatment, falling to the lowest median value of 0.6 k/µl, which is considered Grade 2 lymphopenia. Throughout the recovery period, median ALC values were significantly lower than those of the SBRT alone group, and by the 24-month time point, they had only reached 1.0 k/µl, 67% of the time point 0 value. This suggests a slower and less robust recovery of lymphocyte populations in those treated with supplemental PNI.

The low rate of high-grade lymphopenia with prostate SBRT alone is not unexpected. RRL is believed to be partially due to irradiation of lymphocytes as they transverse the irradiated field, even if it does not include lymphoid tissues such as the bone marrow and/or lymph nodes (3). There is significant prostatic blood flow, and during a single 30- to 40-min SBRT treatment session, the entire blood volume could transverse the prostate. However, only a small percentage (approximately 2%) of overall lymphocytes are circulating in the blood at a given time, while approximately 50% reside in the lymph nodes (17).

Lymphocytes originate from hematopoietic stem cells in the bone marrow (BM), after which they travel to lymph nodes, between nodes, and enter the blood. Homeostasis is the mechanism that prevents lymphocyte depletion in the blood, via the recruitment of lymphocytes from lymphatic organs (18). The pelvic bone is the primary site of hematopoiesis in adults, harboring the majority of proliferating BM and posing as a potential organ at risk when utilizing pelvic IMRT (19).

Given the large treatment volume utilized by pelvic IMRT, identifying optimal dose constraints for pelvic IMRT is necessary to minimize hematologic toxicities and subsequent loss of activity in the bone marrow (19). Circulating lymphocytes may also be irradiated at a higher rate due to the large pelvic treatment volumes that incorporate lymph nodes and large blood vessels. Differences in radiosensitivity between lymphocyte subpopulations impact their relative contributions, likely altering the dynamics of the immune response via changes in lymphocyte diversity and activity (20).

In addition, supplemental PNI is highly fractionated to limit gastrointestinal (GI) toxicities, however, due to the high radiosensitivity of lymphocytes, repeatedly dosing likely increases the risk of high-grade lymphopenia (21–23). Hypofractionation might be an approach to limit both lymphocyte lethality and reduced lymphocyte recruitment to circulating blood following RT (17).

The interpretations of comparable results on RRL post-SBRT in other disease states can be applied to the treatment of prostate cancer in minimizing lymphopenia incidence and grade during and after the course of treatment. In a prospective study, marrow-sparing IMRT was effectively utilized to reduce radiation dose to functional BM in patients with other pelvic malignancies, such as cervical and endometrial cancers (24). This approach was not utilized in this study but could be employed in the future to minimize unintentional dosing of the bone marrow_3_.

Limitations of our study include a smaller number of patients in the SBRT + PNI group and variation in risk group and utilization of ADT between groups, however, disease risk and ADT are not known to cause lymphopenia (25). Additionally, patients were not randomized to treatment groups, and the SBRT + PNI group had lower ALCs at baseline, potentially suggesting prior damage. The authors believe that these confounding variables are unlikely to be responsible for the difference in lymphopenia between the two groups. This study also lacks a measure of the acute effects of SBRT and PNI on ALC shown during or immediately following the treatment course. If we had assessed earlier time points, the nadir would have likely been lower. Finally, we did not have information on how RRL specifically impacted specific lymphocyte subsets. Further characterization of the effects of irradiating lymphoid tissues could provide mechanistic insights into the role of RRL in reducing response to treatment.

Abdominal and pelvic nodal oligometastatic recurrences are common after previous treatment for prostate cancer. Nodal radiation therapy utilizing wider treatment volumes is considered tolerable and effective in disease control; however, the increased risk of lymphopenia associated with this treatment course suggests that smaller treatment volumes could reduce toxicities. SBRT aiming to treat sites with macroscopic evidence of disease as suggested by PSMA-PET has been suggested to be a viable salvage treatment option for both pelvic and para-aortic recurrences (26, 27). The benefit-to-risk ratio should be considered when electing to utilize wide prophylactic volumes such as with PNI, given disease control observed with smaller volumes and decreased risk of toxicities.

Lymphopenia risk can be integrated into radiotherapy treatment planning by utilizing computational tools, such as the HEDOS framework, to estimate the dose to circulating blood cells based on time structure of the treatment and circulation of blood cells through irradiated organs (28). In addition, pelvic bone marrow dose-volume predictors of late lymphopenia following pelvic lymph node RT for prostate cancer, including baseline ALC, could be considered during treatment planning to minimize toxicities proactively (29).

Prostate cancer has been conventionally recognized to be an immunologically “cold” solid tumor, due to its strongly immunosuppressive tumor microenvironment (TME) and low levels of T-cell infiltration and driver mutations (30). Given this phenotype, lymphopenia may not significantly impact prostate cancer progression as it does in other solid tumors. Additionally, immunotherapy has shown reduced efficacy in prostate malignancies due to this tumor profile (31). However, our patient cohort could serve as a potential model system for studying RRL, as patients are generally healthy and have not received previous treatment with chemotherapy or immunotherapy agents. Previously, it has been shown that in patients treated with PD-(L)1 checkpoint inhibitors, prior radiation therapy was strongly associated with lymphopenia at 3 months post-treatment, and patients with lymphopenia had a shorter time to progression (32). Across multiple cancer sites and treatment types, lymphopenia correlates with decreased overall survival, highlighting the importance of minimizing and managing this toxicity in our treatment approaches (33).

## Data Availability

The raw data supporting the conclusions of this article will be made available by the authors, without undue reservation.
