# Supplementary figures and images for: Late radiation-related lymphopenia after prostate stereotactic body radiation therapy plus or minus supplemental pelvic irradiation
Source: Front Oncol. 2024 Nov 21;14:1459732. doi: 10.3389/fonc.2024.1459732 (PMC11617573; doi:10.3389/fonc.2024.1459732)

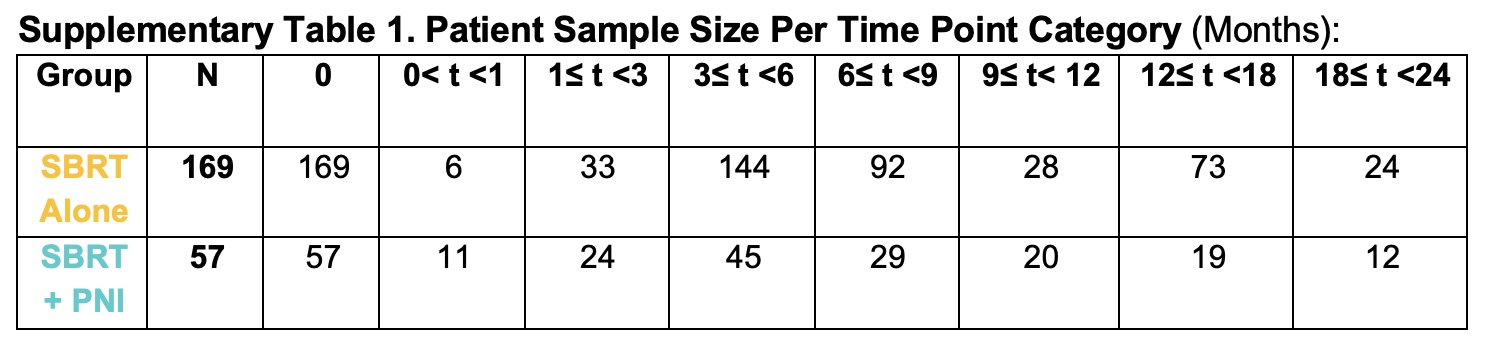

Supplement: Supplementary file 1 [file SupplementaryFile1.jpeg]
